# Is There Value in Pathology Specimens in Routine Total Hip and Knee Arthroplasty?

**DOI:** 10.7759/cureus.13005

**Published:** 2021-01-30

**Authors:** Justin Koss, David Goyette, Jay Patel, Colin J Harrington, Christopher Mazzei, James C Wittig, John Dundon

**Affiliations:** 1 Orthopedics, Morristown Medical Center, Morristown, USA; 2 Orthopedics, Walter Reed National Military Medical Center, Bethesda, USA; 3 Orthopedic Surgery, Orthopedic Institute of New Jersey, Morristown, USA

**Keywords:** pathology, arthroplasty, hip replacement, knee replacement, bundled payments

## Abstract

Background

Routine analysis of bone specimens in total joint arthroplasty (TJA) is mandatory at many institutions. The purpose of this study was to determine if mandatory routine TJA specimen analysis alters patient care or if they represent an unnecessary healthcare expenditure.

Methods

A retrospective review was performed of all primary TJA patients between October 2015 and December 2017 at our institution. Pathology results were reviewed to ascertain the number of concordant, discrepant, and discordant results. A diagnosis was considered concordant if the preoperative and pathologic diagnosis matched, discrepant if the preoperative and pathological diagnosis differed but no change in the patient’s plan of care occurred, and discordant if the preoperative and pathologic diagnosis differed and resulted in a change in the patient’s plan of care.

Results

3,670 total hip arthroplasty (THA) and total knee arthroplasty (TKA) procedures (3,613 patients) met the inclusion criteria and were included in this study. All 3,670 specimens had a concordant diagnosis; there were zero discrepant and zero discordant diagnoses. During the study period, our institution spent $67,246.88 in routine analysis of TJA specimens by a pathologist, with no change in any postoperative patient care plans.

Conclusion

With bundled payment reimbursement models and hospitals trying to decrease unnecessary expenditures, the present study helps further demonstrate that routine analysis has limited cost-effectiveness due to the low prevalence of alteration in the management of patient care. The decision for pathological analysis should be left at the discretion of the surgeon in order to maximize the cost-efficiency of TJA procedures.

## Introduction

Total joint arthroplasty (TJA) is a common procedure in the United States with an annual incidence of 92,780 for total hip arthroplasty (THA) and 156,656 for total knee arthroplasty (TKA), according to the latest American Joint Replacement Registry report (2017) [1]. Annual rates of TJA continue to climb with the rates of THA doubling and rates of TKA tripling between 1990 and 2002 [2-4]. Given our aging population, TJA procedures are expected to increase, with demand for THA expected to grow by 174% and TKA by 673% by 2030 [5]. TJA is also one of the most successful orthopedic operations with one of the highest improvements in quality-adjusted life years (QALY) among all procedures. 

Over the last decade, the cost of healthcare has been increasing exponentially, with TJA accounting for approximately US$ ten billion annually [6]. Currently, THA and TKA account for over US$ 7 billion in costs by the Centers for Medicare and Medicaid Services (CMS) and account for more expenses than any other inpatient procedure [7]. Attempts to curtail costs have led to the creation of Alternative Payment Models (APM). Implementation of the Bundled Payments for Care Improvement Initiative (BPCI), episodes of care, and Comprehensive Care for Joint Replacement (CJR) created a focus on value in medicine. These payment models force hospitals and physicians to cut unnecessary costs while maximizing patient outcomes. The initiation of the BPCI has led to a reduction in the average hospital length of stay, rate of discharge to an inpatient facility, readmission rate, and average cost for TJA procedures [8]. The focus on improved quality, adherence to evidence-based guidelines, and reduction in cost has aligned the interests of physicians and hospital administrators. This has led to increased scrutiny of costs associated with the entire episode of care for TJA. Gainsharing models ensure that physicians have a motive for the savings and losses. Physicians must determine the cost-benefit ratio in order to offer patients the most efficient plan to maximize value in TJA [6].

In many institutions across the country, sending bone removed during routine primary THA and TKA for pathologic analysis is the standard of care and mandated by the hospital. The goal of routine pathologic analysis is to ascertain discrepancies between the histological diagnosis and the physician's preoperative diagnosis with the objective of determining if any changes need to be made in the patient's postoperative care. The purpose of this study was to determine the cost-effectiveness, potential patient care benefit, and the necessity of performing routine pathologic analysis of primary THA and TKA specimens.

## Materials and methods

A retrospective review was conducted of all primary THA and TKA procedures performed at our institution from October 2015 to December 2017. Pathology specimens were obtained from the bone cuts made during TJA surgery. All specimens were stored in formaldehyde, marked by the nursing staff, and transported to the pathology department after the procedure was concluded by a nursing assistant. The final preparation of each specimen was undertaken by the pathology staff and pathologist. The inclusion criterion was all routine primary THA and TKA cases that were performed for degenerative and inflammatory joint conditions such as osteoarthritis, rheumatoid arthritis, and avascular necrosis. Exclusion criterion for the study was any patients undergoing surgery for joint infection, oncologic diagnosis, component removal, and revision TJA cases. The preoperative diagnosis was determined by the operative surgeon prior to the surgery based on their clinical and radiographic evaluation of the patient. ICD9/10 codes were examined and were recorded from the preoperative and postoperative surgeon notes. The pathologic diagnosis, determined via analysis by the pathologist, was recorded as well. All diagnoses were recorded against the pathology report made by the pathologist.

A diagnosis was considered concordant if the preoperative and pathologic diagnosis matched and discrepant if the preoperative and pathological diagnosis differed but no change in the plan of care was observed. A diagnosis was deemed discordant if the preoperative and pathologic diagnosis differed and resulted in a change in the plan of care. Basic statistical analyses were performed using Minitab Software (Mintab, Inc., State College, PA, USA).

## Results

Between October 2015 and December 2017, there were a total of 1,641 THA patients (1,641 THA procedures), with an average age of 66.7 years. During the same study period, there were 1,972 TKA patients (2,029 TKA procedures), with an average age of 69.5 years. The prevalence of concordant results was 100% (3,670 out of 3,670). There were no discrepant results and no discordant results for any of the TJA patients. The total cost spent by our institution on the routine pathological analysis that generated zero discrepant or discordant results during the study period was $67,246.88. A power analysis was performed with alpha set to 5%, beta set to 90%, and the concordance rate set to 99% with an acceptable difference of 1%. 2,144 participants would have been necessary to reach statistical significance.

## Discussion

Value in TJA can be defined by the relationship between quality and cost. Our study found no alteration in care with the use of mandatory routine analysis of pathology specimens in uncomplicated primary TJA. In this study, we found a decrease in value with the mandatory, routine pathologic analysis of specimens in THA and TKA. The total cost spent by our institution on routine pathological analysis during the study period was $67,246.88, approximately $18.32 per TJA procedure. Previous studies have shown that the cost per discrepant diagnosis can range from $4,383 to $4,983 [9,10]. While there have been previous studies reporting similar results for THA and TKA patients, the majority of studies are more than two decades old and many hospitals are still performing this costly test, which brings into question the cost-effectiveness of routine analysis [9-13]. 

Suchman et al. performed a large database review of 630 hospitals across the United States to ascertain how many hospitals are performing a routine histological examination of joint arthroplasty specimens [14]. They found that from 2006 to 2016, the routine histological examination of joint arthroplasty specimens decreased from 34% to 30% for shoulder arthroplasty, from 50% to 45% for THA, and from 43% to 38% for TKA (all p < 0.001). While the numbers are slowly decreasing, there remains a large number of institutions performing routine pathologic analysis. Greene et al. analyzed the cost-effectiveness of routine examination of pathology specimens following knee arthroscopy (partial meniscectomies and anterior cruciate ligament reconstructions) [15]. The prevalence of concordant diagnoses was 99.3%. The total cost per discrepant diagnosis was $13,771, and the cost per discordant diagnosis was $371,810. They concluded that gross and histological examination of the specimen removed during knee arthroscopy should be done at the discretion of the surgeon as opposed to being mandated by hospitals.

The transition from fee-for-service payment models to APMs is resulting in an enhanced quality of care and a reduction in the cost of TJA [16]. In an era where value and cost containment in TJA is paramount, the present study has demonstrated a decrease in value with the routine analysis of pathologic specimens. There were several limitations of our study. The first of which is its retrospective nature and all of the biases associated with a retrospective study. The second limitation is that while the current study includes over 3,600 patients, we did not have any discrepant or discordant results, and therefore, the cost of these findings could not be calculated for our institution. However, the rates of discrepant and discordant diagnoses in the literature have been shown to be exceedingly low, consistent with our findings. It should also be reemphasized that this study was conducted on patients undergoing routine, primary THA and TKA. We excluded all patients that were suspected or confirmed to have an infection or any oncologic diagnoses. Also, the analysis done by the pathologist on the specimens was done postoperatively, and no intraoperative frozen sections were sent.

## Conclusions

Of the 3,670 pathologic specimens in routine TJA, the data demonstrate that mandatory routine pathologic analysis offered no alteration in patient care while increasing the cost of care. There are still a large number of hospitals across the nation mandating pathological analysis of specimens in routine orthopedic surgery, though there are no formal guidelines or substantial evidence to support this practice. With the changing landscape of reimbursements to bundled care models, the present study helps further demonstrate that mandatory analysis of routine primary THA and TKA specimens by a pathologist is not cost-effective due to the low prevalence of alteration in the management of patient care. The decision for pathological analysis should be left to the discretion of the surgeon in order to maximize the cost-efficiency of THA and TKA procedures.
